# Retraction: SPAG6 hypermethylation silences a novel tumor suppressor and inhibits renal cell carcinoma progression via PI3K/AKT/mTOR pathway

**DOI:** 10.1371/journal.pone.0349920

**Published:** 2026-05-22

**Authors:** The PLOS One Editors

The *PLOS One* Editors retract this article [1,2] due to concerns about potential manipulation of the publication process. These concerns call into question the validity and provenance of the reported results. We regret that the issues were not identified prior to the article’s publication.

TW and QZ did not agree with the retraction. XJ, YY, XW, YG, and YF either did not respond directly or could not be reached.
